# Application of ThinPrep Bronchial Brushing Cytology in the Early Diagnosis of Lung Cancer: A Retrospective Study

**DOI:** 10.1371/journal.pone.0090163

**Published:** 2014-04-23

**Authors:** Chaoying Liu, Zhongmei Wen, Yang Li, Liping Peng

**Affiliations:** Department of Respiratory Medicine, The First Hospital of Jilin University, Changchun, China; Kliniken der Stadt Köln gGmbH, Germany

## Abstract

The majority of lung cancer patients are diagnosed at advanced stages of disease. This study evaluated the diagnostic value of ThinPrep (TP) bronchial brushing cytology in lung cancer. A total of 595 patients with suspicious lung cancer were enrolled in this study. The bronchial brushing samples were prepared by TP. The data were then compared to histology of lung tissue samples. Histologically, 479 of these 595 patients were diagnosed with lung cancer, including 223 cases of lung squamous cell carcinoma (SCC), 77 cases of lung adenocarcinoma (ADC), and 152 cases of small cell lung carcinoma (SCLC). The TP cytology revealed a total of 460 cases of lung cancer (including 232 SCCs, 91 ADCs, and 108 SCLCs). The TP cytological technique had 87.06% sensitivity and 62.93% specificity in the diagnosis of lung cancer. Specifically, TP cytology confirmed 195 of 223 SCCs, 47 of 77 ADCs, and 94 of 152 SCLCs. The TP cytology showed 87.44% sensitivity and 90.05% specificity for the diagnosis of SCC, with a Matthew's correlation coefficient (MCC) of 0.820; while the sensitivity was reduced to 61.04% and the specificity was 90.93% for the diagnosis of ADC, with a MCC of 0.464. For the diagnosis of SCLC, the sensitivity was 61.84% and the specificity was 96.84%, with a MCC of 0.648. Thus, this study demonstrated the usefulness of TP bronchial brushing cytology in the early diagnosis of lung cancer, especially the early stage of lung SCC. A prospective clinical trial will verify these data before being translated into the clinic.

## Introduction

Lung cancer is the most significant worldwide health problem and contributes to 30% of male and 26% of female cancer-related deaths, and approximately 1.38 million people die of lung cancer each year [1], [2]. To date, most lung cancer patients are diagnosed at an advanced stage of disease, making curable surgery not an option; thus, early diagnosis and effective treatment are key to prolong the survival of lung cancer patients. To this end, our research utilizes cytology of bronchial brush specimens for the initial evaluation and diagnosis of lung cancer. There are a variety of sample preparation techniques available for cytological evaluation and detection of lung cancer [3], including exfoliative and abrasive cytology (bronchioalveolar lavage, bronchial brushing, or bronchial washing) and fine needle aspiration (FNA) cytology [4]–[6]. Nevertheless, the liquid-based, monolayer cytological preparation (ThinPrep, TP) is an automated cytopreparatory technique that has been designed to improve both sample collection and cytopreparation [7]. The utility of TP of lung cell samples has been widely accepted as a major diagnostic method in various clinical settings and has been proven to have a great association with tissue histological diagnosis [8]–[11]. The advantages of this method include improved visualization of diagnostic cells; uniform thickness of cytology slides, better cellular preservations, removal of air-drying artifacts, and elimination of obscuring blood and inflammatory exudate [12]. However, it does have a disadvantage; for example, malignant cells inside the hemorrhagic body-cavity effusions could be easily visualized as tissue fragments and may be removed by the TP processor. This weakness may decrease the sensitivity of TP and underscore its importance as a useful diagnostic tool. But improper sampling preparations could easily compromise the quality of the cytology slide due to air-drying, a thick smear, or other damage to the cell morphology [7]. Thus, TP bronchial brushing cytology may provide an initial diagnosis of lung cancer. Previous studies have shown that TP has been frequently used in the diagnosis of lung cancer by using cytological samples from sputum, bronchial washing, or FNA [6], [8], [13]. To date, there have been very few studies that use TP bronchial brushing samples. Thus, the aim of the current study was to compare the diagnostic value of TP bronchial brushing cytology in lung cancer to the corresponding histological diagnosis in each of 595 patients with suspicious lung cancer who underwent a fibro bronchoscopy.

## Materials and Methods

### Patient Population and Sample Preparation

A total of 595 consecutive bronchial brushing cases were retrospectively enrolled from the First Hospital of Jilin University (Changchun, China) between May 2010 and May 2012. These patients included 381 males and 214 females with an average age of 57 years old for males and 55.4 years old for females (Table 1). This study was approved by the Ethical Committee of the 1st Hospital of the Jilin University. All participants gave written consent to participate in this research and the details of this consent procedure. Written consent was given by the patients for their information to be stored in the hospital database and used for research.

**Table 1:** Patient Demographics.

Age (years)	20–30	31–40	41–50	51–60	61–70	71–80	81+	Total	Average Age
Male	10	14	75	137	112	35	0	381	57±10.69
Female	6	13	44	80	53	16	1	214	55.4±11.42
Total	16	27	119	217	165	51	1	595	56.42±10.98

Note: A total of 595 consecutive bronchial brushing cases were included in this study with 381 male and 214 female patients.

Two cytopathologists, who were experienced lung cytologists in our institution, analyzed the samples. The bronchial washing samples were obtained during fiberoptic bronchoscopy procedures performed in patients with a clinical or radiological diagnosis of a suspected pulmonary neoplasm. Bronchial washing specimens were collected in CytoLyt collection media. All specimens were prepared by the TP method (Cytyc Corp., Marlborough, MA) according to the manufacturer's recommended procedures. The bronchial mucus biopsy was simultaneously conducted for all cases. The cytological diagnosis followed the World Health Organization (WHO) classification guidelines [14]. The staging of non-small cell lung carcinoma (NSCLC) was defined according to the guidelines of the 2009 International Association for the Study of Lung Cancer (IASLC). The staging of small cell lung carcinoma (SCLC) was defined according the Veterans Administration Lung Study Group staging system. SCLC was divided into limited stage (LS) and extensive stage (ES). The histological diagnosis of the bronchial biopsy was considered as the patient's final diagnosis. Out of these 595 patients, tissue histology diagnoses showed 116 benign cases and 479 cases of lung cancer. In the lung cancer cases, there were 223 squamous cell carcinomas (SCCs), 77 adenocarcinomas (ADCs), and 152 small cell carcinomas (SCLCs).

### TP Specimen Preparation and Processing

For TP specimen preparation and processing, all bronchial washing specimens were placed in 30 ml of CytoLyt, centrifuged, resuspended in PreservCyt Solution (Xinbaishi, USA), and put onto the TP processor with one slide per specimen. The slides were then stained with Papanicolaou stain and covered with a coverslip. The cytology slides were then blindly examined under a microscope independently by two cytopathologists. The evaluations of cytomorphological features included overall cellularity, preservation of cellular morphology, and removal of background materials (e.g., inflammatory or blood cells, mucus). All cytology slides were assigned to one of the following five categories: (A) Unsatisfactory: there were no or few epithelial cells and macrophages with an obscure background (e.g., inflammation, blood, and mucus); (B) Normal: there were only normal cellular components; (C) Benign: there were normal cellular components with inflammatory cells but without atypical cells; (D) Suspicious: there were atypical cells that were suspicious of malignancy; (E) Malignant: there were definite malignant cells.

### Statistical Analysis

The TP cytological data were compared to the histological diagnoses and evaluated for the sensitivity, specificity, positive predictive value (PPV), and negative predictive value (NPV). The Matthews correlation coefficient (MCC) [29], [30] was calculated to determine the correlation of the TP cytological diagnosis with the tissue histological diagnosis. The MCC ranges between −1 and +1. A coefficient of +1 represents a perfect prediction, 0 denotes no better than a random prediction, and −1 indicates total disagreement between the prediction and observation.

## Results

### Patient Characteristics

A total of 595 consecutive bronchial brushing cases were retrospectively evaluated in this study, which included 381 male and 214 female patients. The bronchial washing specimens were obtained during fiberoptic bronchoscopy procedures. The average age of patients in this study was 56.42 years old (57 years for men and 55.4 years for women). The age of patients diagnosed with lung cancer mainly ranges between 50 to 70 years old in China; thus, there was no significant difference in terms of age for both male and female patients in this study. However, lung cancer occurs more often in men than in women. The patient demographics are summarized in Table 1. The tumor stages of the patients with SSC, ADC, or SCLC are summarized in Table 2. The majority of patients with SSC were in the early stage, whereas the majority of patients with ADC were in the advanced stage. Moreover, 58.6% of patients with SCLC were in the limited stage, and 41.4% of them were in the extensive stage.

**Table 2:** Association of clinical stages of lung cancer with ThinPrep.

Stage	N (%)	ThinPrep (% of positive cases)	P value
Ia	19 (8.5)	84.2	
Ib	55 (24.7)	87.3	
IIa	32 (14.3)	90.6	
IIb	47(21.1)	93.6	
IIIa	36 (16.1)	86.1	
IIIb	24 (10.8)	83.3	
IV	10 (4.5)	70	
Total	223 (100)		0.505
Ia	0 (0)	0	
Ib	7 (9.1)	42.9	
IIa	2 (2.6)	50	
IIb	9 (11.7)	44.4	
IIIa	23 (29.9)	60.9	
IIIb	17 (22.1)	70.6	
IV	19 (24.7)	68.4	
Total	77 (100)		0.668
Limited	89 (58.6)	68.6	
Extensive	63 (41.4)	52.4	
Total	152 (100)		

SCC, squamous cell carcinoma; ADC, adenocarcinoma; SCLC, small cell lung carcinoma.

### Cytomorphological Characteristics of Lung Cancer in TP Cytology

**Figure 1:**
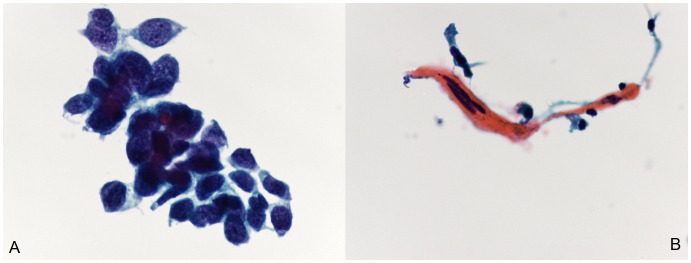
Cytomorphology of lung squamous cell carcinoma (SCC) using ThinPrep bronchial brushing cytology. Small clusters of malignant cells with features of poorly differentiated carcinoma. (A), Well-differentiated SCC. Cytological features are characterized by individual cells or cohesive sheets of tumor cells with clear cell borders and a dense cytoplasm. (B), Poorly differentiated SCC. Cytology shows high cellularity and small groups of tumor cells. The nuclear/cytoplasmic (N/C) ratio was usually high (Papanicolaou staining; the original magnification was 400×).

**Figure 2:**
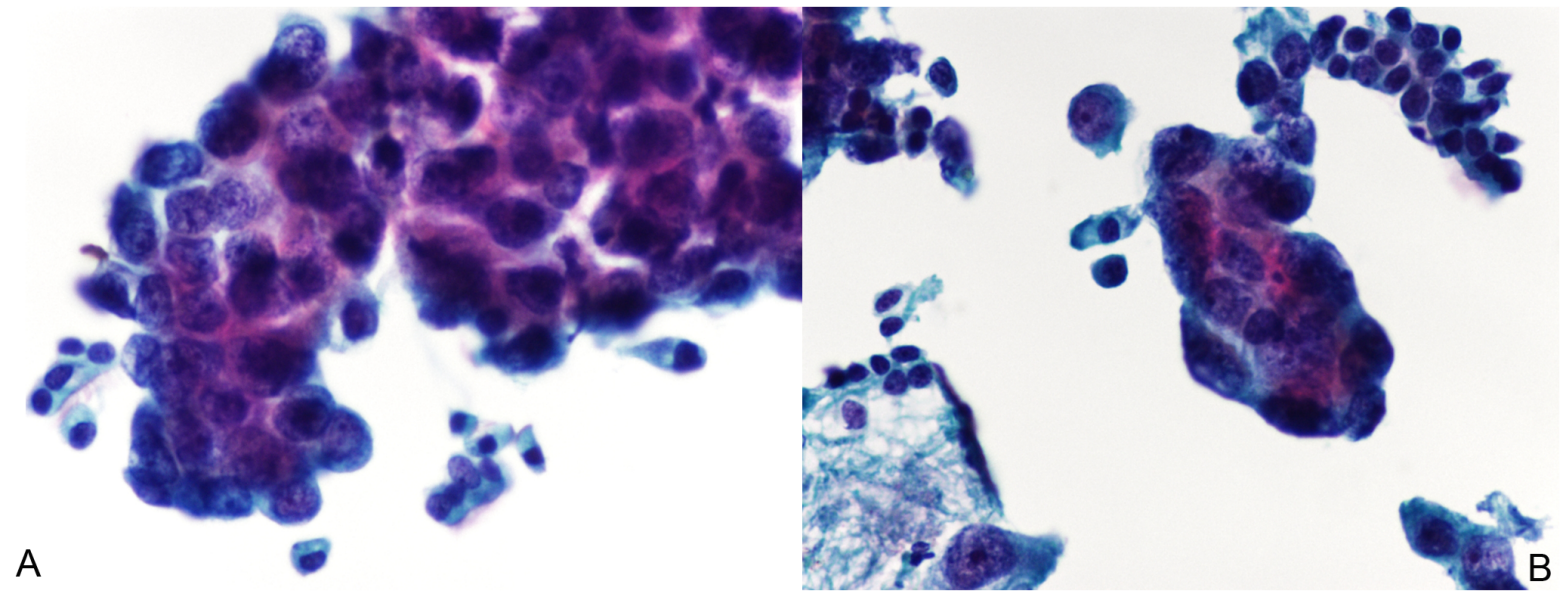
Cytomorphology of lung adenocarcinoma (ADC) in ThinPrep bronchial brushing cytology. (A and B), Typical features of ADC in cytology include cellular clusters with a deep focus due to the three-dimensional clusters of large vacuolated cells. Sometimes, the individual cells or acinar (glandular) arrangements were visible in the cytology slides. The nuclear/cytoplasmic (N/C) ratio was usually high (Papanicolaou staining; the original magnification was 400×).

**Figure 3:**
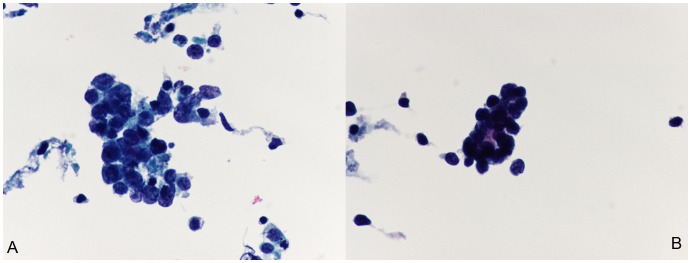
Cytomorphology of small cell lung carcinoma (SCLC) in ThinPrep bronchial brushing cytology. (A and B), The features of SCLC in cytology included a biphasic population of viable and degenerating tumor cells. These cells had a scant cytoplasm with a thin cyanophilic rim, single or loose clusters, molding, and DNA artifacts. The nuclear/cytoplasmic (N/C) ratio was usually high (Papanicolaou staining; the original magnification was 400×).

Morphologically, the characteristics of the three major subtypes of lung cancer presented differently. In brief, SCC is usually a centrally localized tumor that grows inside of the bronchus or the lung; thus, it is relatively easy to obtain a large amount of tumor cells in bronchial brushing samples. The TP cytology features of well-differentiated SSC were characterized by individual cells or cohesive sheets of tumor cells with clear cell borders and a dense cytoplasm, while poorly differentiated SCC was difficult to distinguish from poorly differentiated ADC. Typically, it was seen as high cellularity and small groups of tumor cells. The nuclear/cytoplasmic (N/C) ratio was relatively high (Figure 1A and 1B). Furthermore, ADC is usually located in a peripheral portion of the lung. Typical features of ADC in TP cytology included cellular clusters with a deep focus because of the three-dimensional clusters of large vacuolated cells. Sometimes, the individual cells or acinar (glandular) arrangements were visible in the TP cytology slides. The N/C ratio was usually high (Figure 2A and 2B). In addition, SCLC is usually localized centrally. SCLC is a high-grade neuroendocrine tumor and is associated with tobacco smoke and a poor prognosis. The features of SCLC in TP cytology included a biphasic population of viable and degenerating tumor cells. These cells had a scant cytoplasm with a thin cyanophilic rim, single or loose clusters, molding, and DNA artifacts. The N/C ratio was high (Figure 3A and 3B).

### Association of TP Cytology with Tissue Histological Diagnosis

The final tissue histology of bronchial biopsies confirmed 479 lung cancer cases from these 595 patients. Particularly, out of these 479 patients, there were 223 SCCs, 77 ADCs, and 152 SCLCs. The TP bronchial brushing cytological data showed 460 lung cancer cases, out of 595 patients (i.e., 232 SCCs, 91 ADCs, and 108 SCLCs). Therefore, the TP cytology successfully diagnosed 417 cases out of 479 lung cancers, the diagnosis sensitivity was 87.06%, the diagnosis specificity was 62.93%, the PPV was 90.65%, the NPV was 54.07%, and the MCC was 0.473. However, according to the lung histology, the TP cytology successfully detected 195 SCCs out of 223 cases, 47 ADCs out of 77 cases, and 94 SCLCs out of 152 cases. The sensitivity of TP cytology for the diagnosis of SCC was 87.44%, the specificity was 90.05%, the PPV was 84.05%, the NPV was 92.29%, and the MCC was 0.820. In contrast, the sensitivity for ADC diagnosis was reduced to 61.04% and the PPV to 50.00%, but the specificity was still high (90.93%), the NPV was 94.01%, and thus the MCC was reduced to 0.464. Similarly, the sensitivity for SCLC diagnosis was 61.84%, the specificity was 96.84%, the PPV was 87.04%, the NPV was 88.09%, and the MCC was 0.664 (Table 3).

**Table 3:** Association of ThinPrep Bronchial Brushing Cytology with Lung Cancer Histology.

		Histological Diagnosis (No. of Cases)							
		Positive	Negative	Total	SEN (%)	SPE (%)	PPV (%)	NPV (%)	MCC
LC	Positive	417	43	460	87.05	62.93	90.65	54.07	0.473
	Negative	62	73	135					
	Total	479	116	595					
SSC	Positive	195	37	232	87.44	90.05	84.05	92.29	0.820
	Negative	28	335	363					
	Total	223	372	595					
ADC	Positive	47	47	94	61.04	90.93	50.00	94.01	0.464
	Negative	30	471	501					
	Total	77	518	595					
SCLC	Positive	94	14	108	61.84	96.84	87.04	88.09	0.664
	Negative	58	429	487					
	Total	152	443	595					

Note: LC, lung cancer; SCC, squamous cell carcinoma; ADC, adenocarcinoma; SCLC, small cell lung carcinoma; SEN, sensitivity; SPE, specificity; PPV, positive predictive value; NPV, negative predictive value; MCC, Matthews correlation coefficient.

## Discussion

In this study, we assessed the diagnostic value of TP bronchial brushing cytology in lung cancer in comparison to the corresponding histological diagnosis in each of 595 patients with suspicious lung cancer who underwent a fibro bronchoscopy. We found that compared to the histological diagnosis by bronchial biopsy, TP bronchial brushing cytology was closely associated with the corresponding histological diagnosis, suggesting that this method is very useful to add to lung cancer diagnosis, especially for SCC with more than 90% sensitivity and specificity. Moreover, the MCC was up to 0.820, which translates into an excellent agreement with the histological diagnosis of SCC. However, a prospective clinical trial is needed to verify this finding.

In our current study, we found that patient demographic data showed that the prevalence of lung cancer predominantly affected those aged from 50 to 70 years old in China, which is consistent with data from other countries [15], [16]. But there was no significant preference between men and women in terms of age. The average age was 56.42 years old in this cohort of patients. Thus, TP bronchial brushing cytology may apply to this age group for screening lung cancer. Indeed, respiratory cytology is increasingly being used in the initial evaluation of pulmonary disorders, particularly, in lung cancer [6], [8], [13], [17]–[19]. Bronchial brushing is often obtained during fiberoptic bronchoscopy along with bronchial washing or transbronchial FNA/forcep biopsies [6]. Thus, TP cytology can be utilized in these samples for the early diagnosis of lung cancer. In the current study, we obtained bronchial brushing samples from fiberoptic bronchoscopy and utilized the TP sample preparation procedure in a total 595 patients who were suspected of having lung cancer. Out of these 595 patients, 479 cases were histologically diagnosed as having lung cancer, while TP cytology diagnosed 460 cases of lung cancer. Compared to the histological diagnosis, TP cytology had a sensitivity of 87.06% and a specificity of 62.93% for lung cancer diagnosis. Using the lung cancer histological breakdown, TP bronchial brushing cytology had an even higher sensitivity and specificity for lung SCC diagnosis, indicating that this method is useful in the early detection of lung cancer, especially when the histological diagnosis is not available (such as nonresectable lung cancer).

Furthermore, the precise subclassifications of lung cancer are critical for the effective management of patients [20]. A false classification will result in delayed treatment and lead to high mortality. This is specifically applicable for advanced lung cancer patients with unresectable disease. Clinicians will utilize cytology as a means to make the diagnosis as accurate as possible. Thus, TP bronchial brushing cytology or TP transbronchial FNA cytology could be used to assist lung cancer diagnosis with confirmation of histology type. In our current study, we further grouped patients into those having SCC, ADC, and SCLC based on TP cytology. The TP cytology confirmed that 195 of 223 patients had SCC, 47 of 77 had ADC, and 94 of 152 had SCLC. The diagnostic value of the TP cytology was the highest in those with SCC among these three different histological types of lung cancer. This finding implies that TP bronchial brushing cytology is more beneficial to the diagnosis of early stage lung SCC, but less beneficial to the diagnosis of ADC or SCLC.

The reason for this result may be because SCC is usually a centrally localized tumor and is easy to obtain a great number of tumor cells from the bronchial brushing. In contrast, ADC is generally located in a peripheral portion of the lung and it is more difficult to obtain sufficient numbers of tumor cells either by bronchial brushing or bronchial biopsy. Thus, using TP cytology results in a lower sensitivity and specificity in detecting ADC compared to SCC. In addition, in our current study, the sensitivity of TP cytology in detecting SCLC was much less than that in SCC, but similar to that in ADC. A previous study showed that detection of SCLC on TP sputum cytology was less than detecting SCC or ADC; the PPV of TP cytology in the cytodiagnosis of SCCL was 97.5%, and the sensitivity was 62.8%. This finding is very similar to our current study on SCLC. However, a differential diagnosis of SCCL from other lung tumors has important clinical and therapeutic implications [18].

In addition, the TP cytology samples in our current study were obtained from the bronchial brushing during the fibro bronchoscopic examination of patients with suspicious lung cancer. To the best of our knowledge, few studies have been conducted using bronchial brushing samples in TP cytology for the diagnosis of lung cancer. Most TP cytology studies have utilized samples from sputum, bronchial washing, or FNA [8], [21], [22]. The majority of studies compared TP cytology with conventional sample preparation (CP). These comparative studies showed that TP cytology was better than CP in nongynecological cytology [8]–[12], [23], [24]. For example, Rana and colleagues reported that the TP method was superior to CP in terms of cellularity and cytomorphology, and was associated with a reduced unsatisfactory rate and a slightly increased diagnostic rate [11]. Elsheikh and colleagues [9] showed that TP cytology was superior to cytospin sample preparation in the majority of cases during the evaluation of exfoliative cytology specimens, suggesting that TP cytology was more useful and cost effective than cytospin.

In future studies, we will evaluate the TP cytological technique using other samples, such as sputum, which is a noninvasive test for the initial diagnosis of respiratory disorders. Previous studies have shown that the sensitivity of this sample is much less than other samples [8], [25], [26]. For example, Choi and colleagues compared 955 sputum specimens that were prepared by both TP and CP. They found that the preservation of nuclear details was much better on the TP slides than that on the CP slides. The false-negative rates were 49.6% (TP) and 69.4% (CP) in the diagnosis of lung cancer [8]. In our current study, the false-negative rate in evaluating lung cancer was consistent with this study [9]. Furthermore, CT-guided FNA is also a widely used technique in the initial assessment of a patient with suspicious cancer [10], [27], [28]. It has been reported that TP cytology of FNA samples had a high overall diagnosis rate for malignant lung lesions. The sensitivity, specificity, PPV, and NPV were 93.59%, 100%, 100%, and 28.58%, respectively, whereas they were 69.23%, 100%, 100%, and 7.69%, respectively, using the CP diagnostic technique [10]. Thus, further studies are needed to assess different sampling methods for TP cytology in the early diagnosis of lung cancer.

However, there were certain limitations in this study. For example, our data could be even stronger if they were compared with other biomarkers. However, due to detection limitations in our hospital, we were unable to do so. Moreover, this study was initially designed to determine the significance of the TP technique and we did not collect survival data from the patients for association. Our current data did show the usefulness of TP bronchial brushing cytology in the early diagnosis of lung cancer, especially lung SCC. Thus, a prospective clinical study will verify this finding and then translate it into clinical practice.
